# Electrochromism in Electropolymerized Films of Pyrene-Triphenylamine Derivatives

**DOI:** 10.3390/polym11010073

**Published:** 2019-01-05

**Authors:** Tian-Ge Sun, Zhi-Juan Li, Jiang-Yang Shao, Yu-Wu Zhong

**Affiliations:** 1 Key Laboratory of Photochemistry, Beijing National Laboratory for Molecular Sciences, CAS Research/Education Center for Excellence in Molecular Sciences, Institute of Chemistry, Chinese Academy of Sciences, Beijing 100190, China; suntiange@iccas.ac.cn (T.-G.S.); lizhijuan@iccas.ac.cn (Z.-J.L.); shaojiangyang@iccas.ac.cn (J.-Y.S.); 2 School of Chemical Sciences, University of Chinese Academy of Sciences, Beijing 100049, China

**Keywords:** electropolymerization, electrochromism, electrochemistry, triphenylamine, near-infrared

## Abstract

Two star-shaped multi-triphenylamine derivatives **1** and **2** were prepared, where **2** has an additional phenyl unit between a pyrene core and surrounding triphenylamine units. The oxidative electropolymerization of **1** and **2** occurred smoothly to give thin films of polymers **P1** and **P2**. The electrochemistry and spectroelectrochemistry of **P1** and **P2** were examined, showing two-step absorption spectral changes in the near-infrared region. The electrochromic properties, including contrast ratio, response time, and cyclic stability of **P1** and **P2** were investigated and compared. Thin film of **P2** displays slightly better electrochromic performance than **P1**, with a contrast ratio of 45% at 1475 nm being achieved.

## 1. Introduction

Electrochromic materials show reversible color and absorption spectral changes driven by external potential or current stimulus [1]. They have a wide range of applications including smart window [2], information storage [3], and digital displays [4]. Compared to electrochromic inorganic oxides [5] and metal complexes [6], organic semiconductor polymers have shown their appealing advantages in electrochromism, such as easy processability, good flexibility, low cost, and the great capability to adjust color and absorption wavelength through chemical modifications [7,8].

Beyond the commonly-studied electrochromism in the visible region, electrochromic materials in the near infrared (NIR, 750–3000 nm) region have been recently developed and applied in optic fiber telecommunications and dynamic camouflage [9,10]. For instance, Liou and Hsiao and co-workers have demonstrated excellent NIR electrochromic properties using triarylamine-based polymers [11,12,13]. Wang and Wan and co-workers reported appealing NIR electrochromism with good contrast ratio based on thin films of diruthenium complexes [14,15]. We have used multi-center ruthenium compounds as the active materials for NIR electrochromism [16,17]. In this context, the development of NIR electrochromic films has received continued interest in term of easy processability and good electrochromic properties [18,19].

**Figure 1:**
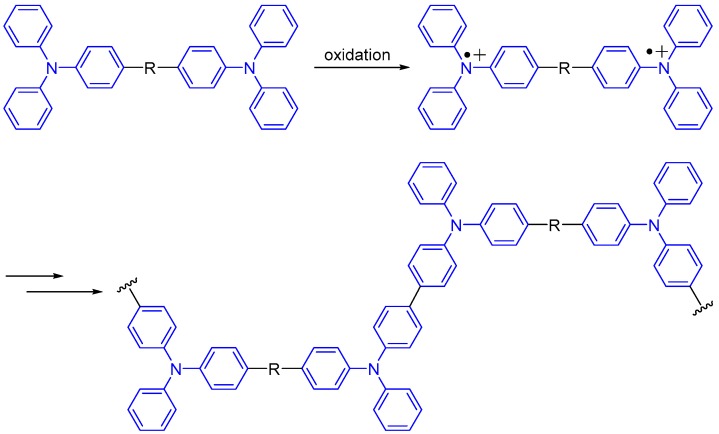
Schematic representation of the electropolymerization of triphenylamine monomers. R stands for any building block between two triphenylamine units.

Drop casting, spin coating, self-assembly [20], and electropolymerization are representative methods to prepare thin films of functional materials [21]. Among them, electropolymerization stands out as a convenient method to achieve polymer formation and film deposition simultaneously. This decreases the experimental time and cost and partially solves the solubility problem of some compounds. A large number of electropolymerized films have been prepared from monomers functionalized with thiophene or carbazole derivatives [22,23,24,25,26,27,28]. In addition, triphenylamine groups have been used as polymerizable units to prepare thin films of organic and metal complexes by electropolymerization [29,30,31,32]. Triphenylamine can be electrochemically oxidized into aminium cation radical, which acts as the key intermediate to form polymeric structures connected by tetraphenylbenzidine bridges (Figure 1) [29,30,31,32]. Interestingly, these newly-generated tetraphenylbenzidine units are electroactive and show strong NIR absorptions as a result of the intervalence charge transfer (IVCT) transitions in the mixed-valent state [33,34,35]. However, the use of this property in NIR electrochromism has not been fully exploited.

**Scheme 1:**
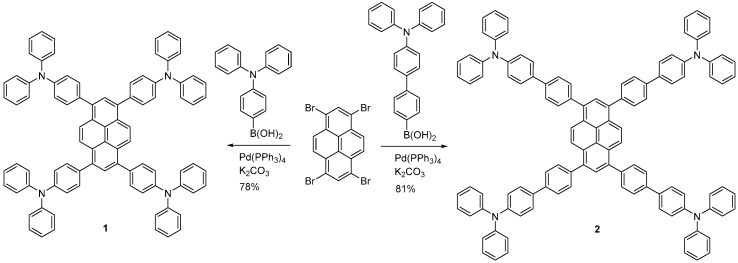
Synthetic routes of compounds **1** and **2**.

We present, herein, two star-shaped compounds **1** and **2** with a pyrene core functionalized with four triphenylamine units for the preparation of NIR-electrochromic thin films by electropolymerization (Scheme 1). The electrochromic properties of the resulting polymers **P1** and **P2** are discussed. Pyrene derivatives are well-known organic semiconductors with interesting optoelectronic properties [36,37,38]. The combination of pyrene with triphenylamine polymers is expected to yield materials with appealing electrochromic properties. Compounds **1** and **2** have different length of the side arms. They are used to examine the structure-property relationship of the resulting polymeric films.

## 2. Materials and Methods

### 2.1. Materials and Instruments

4′-(Diphenylamino)-1,1′-biphen-4-ylboronic acid was prepared according to the known procedure [39]. Other chemicals and reagents were purchased from commercial sources. Details of characterization methods of new compounds are provided in our previous reports [16,35].

### 2.2. Synthesis of Compounds

#### 2.2.1. Compound **1**

A mixture of 4-(diphenylamino)phenylboronic acid (144.6 mg, 0.50 mmol), 1,3,6,8-tetrabromopyrene (51.8 mg, 0.10 mmol), [Pd(PPh_3_)_4_] (9.3 mg, 0.0080 mmol), 2 mL of aqueous K_2_CO_3_ solution (2.0 M) and 5 mL of THF was refluxed at 90 °C for 24 h. The product precipitated out from the solution and was collected by filtration and washing with copious dichloromethane. The product **1** (92 mg) was obtained as a yellow solid in 78% yield. ^1^H NMR (400 MHz, CDCl_3_) δ 8.29 (s, 4H), 8.03 (s, 2H), 7.55 (d, *J* = 8.2 Hz, 8H), 7.31 (t, *J* = 7.7 Hz, 16H), 7.22 (t, *J* = 5.7 Hz, 24H), 7.06 (t, *J* = 7.2 Hz, 8H) (Figure S4). MALDI-HRMS calcd. for C_88_H_63_N_4_ [M + H]^+^: 1175.50526. Found: 1175.49990 (Figure S6). ^13^C NMR data has not been recorded due to the limited solubility of the sample.

#### 2.2.2. Compound **2**

A mixture of 4′-(diphenylamino)-1,1′-biphen-4-ylboronic acid (96 mg, 0.26 mmol), 1,3,6,8-tetrabromopyrene (27 mg, 0.052 mmol), [Pd(PPh_3_)_4_] (4.8 mg, 0.0041 mmol), and 1 mL of aqueous K_2_CO_3_ (2.0 M) and 3 mL of THF was refluxed at 90 °C for 24 h. Product **2** was purified using the same method for the synthesis of **1**. In this synthesis, 63 mg of **2** was obtained as a yellow solid yield in 81% yield. ^1^H NMR (400 MHz, CDCl_3_) δ 8.29 (s, 4H), 8.11 (s, 2H), 7.76 (s, 16H), 7.60 (d, *J* = 8.5 Hz, 8H), 7.27 (16H), 7.18 (t, *J* = 8.3 Hz, 24H), 7.05 (t, *J* = 7.3 Hz, 8H) (Figure S5). MALDI-HRMS calcd. for C_112_H_79_N_4_ [M + H]^+^: 1479.60346. Found: 1479.62625 (Figure S7). Due to the limited solubility of the sample, ^13^C NMR data has not been recorded.

### 2.3. Electrochemistry

The details and equipment information for electrochemical measurements could be found in our previous publications [16,35].

### 2.4. Spectroelectrochemical Measurements

The spectroelectrochemical measurements were performed in 0.1 M of *^n^*Bu_4_NClO_4_/ClCH_2_CH_2_Cl. The polymeric film on the indium–tin-oxide (ITO) glass electrode was used as the working electrode. Other information on the spectrophotometer and potentiastat has been disclosed [16,35]. 

## 3. Results

### 3.1. Synthesis of Monomers

Compounds **1** and **2** were synthesized in good yield by the Suzuki coupling reaction of 1,3,6,8-tetrabromopyrene with 4-(diphenylamino)phenylboronic acid or 4′-diphenylamino-1,1′-biphen-4-ylboronic acid (Scheme 1). These two compounds had a rather low solubility and precipitated out from the reaction mixture when the reaction was complete. A simple filtration procedure led to the isolation of the product, which was used as the monomer for the following electropolymerization experiments. The characterization data of these monomers are described in detail in the Supplementary Materials.

### 3.2. Electropolymerization

**Figure 2:**
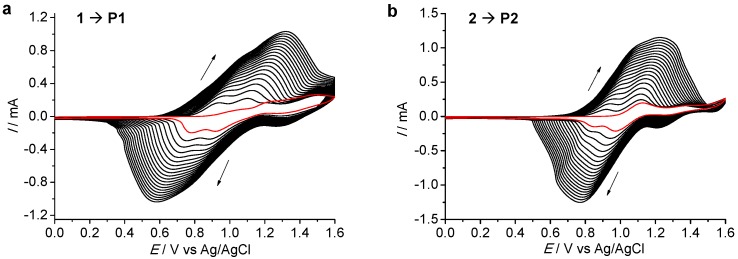
Cyclic voltammograms (CVs) recorded during the oxidative electropolymerization of (**a**) **1** and (**b**) **2** on an indium–tin-oxide (ITO) glass electrode by 20 repeated potential scan cycles between 0 and +1.6 V at 100 mV/s. The plots in red color are the first cyclic scan.

Compounds **1** and **2** were readily electropolymerized on indium−tin-oxide (ITO) glass electrodes by repetitive cyclic voltammetric (CV) scan (Figure 2). The experiments were carried out in 0.1 M *^n^*Bu_4_NClO_4_ in a 1:1 mixture of 1, 2-dichloroethane and chlorobenzene at a scan rate of 100 mV/s. Chlorobenzene was used to increase the solubility of monomers. Taking monomer **1** as an example, the occurrence of the polymerization was supported by the continuous increase of the current upon cyclic potential scans between 0 and +1.6 V vs. Ag/AgCl (Figure 2a). The appearance of two new redox waves at +0.86 and +1.00 V (half-wave potential, *E*_1/2_) was observed during the reverse scan and the following positive scans. These two waves are assigned to the stepwise oxidations of the newly-formed tetraphenylbenzidine units in the polymeric structures. Similar new redox waves were recorded in the electropolymerization of monomer **2** (Figure 2b). Electropolymerization also occurred when the potential scan was reversed at a potential between +1.2 and +1.4 V (Figure S1). However, judging from the degree of current increase, the polymerization process is relatively slower compared to that scanned between 0 and +1.6 V. The obtained polymers **P1** and **P2** have very low solubility, which inhibits their further analysis by NMR or mass spectra. The degree of polymerization of **P1** and **P2** is not known at this stage. The polymer **P1** film was examined by scanning electron microscope (SEM) analysis, which showed that the film adhered tightly to the ITO substrate (Figure S2). The film’s thickness was estimated to be around 50 nm. The polymer **P2** film has a very similar morphology and thickness.

**Figure 3:**
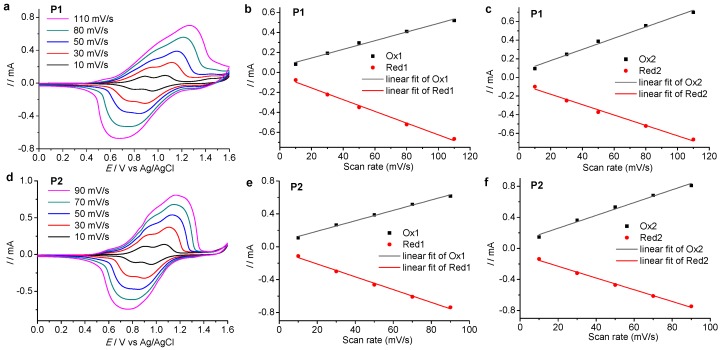
CVs obtained at different scan rates of (**a**) **P1** (10, 30, 50, 80, and 110 mV/s, respectively) and (**d**) **P2** (10, 30, 50, 70, and 90 mV/s, respectively). Linear dependence of peak currents of (**b**,**e**) the first redox wave and (**c**,**f**) the second redox wave of (**b**,**c**) **P1** and (**e**,**f**) **P2** as a function of scan rate. Ox1, Red1, Ox2, Red2 stand for the first oxidation, first reduction, second oxidation, and second reduction peak, respectively. The *R*^2^ values are over 0.98 for all linear fit. The polymeric films on ITO glass electrode were used as the working electrode.

Figure 3 shows the CVs of the above-obtained polymeric films **P1** and **P2** on ITO glass in clean electrolyte solution (0.1 M *^n^*Bu_4_NClO_4_ in dichloroethane). Both polymers exhibited two redox waves at +0.86 and +1.00 V, which were well separated at a slow scan rate of 10 mV/s. As the scan rate increased, the potential separation between two redox couples became less-defined. However, a linear dependence of both anodic and cathodic currents of two redox couples as a function of the scan rate could be derived. This indicates that the redox events are controlled by the surface-confined electron transfer kinetics rather than diffusion process. On the basis of the charge integration associated with these redox waves, the electrochemical surface coverage (*Γ*_echem_) of both films was estimated to be around 7.0 × 10^−9^ mol/cm^2^.

### 3.3. Spectroelectrochemistry

**Figure 4:**
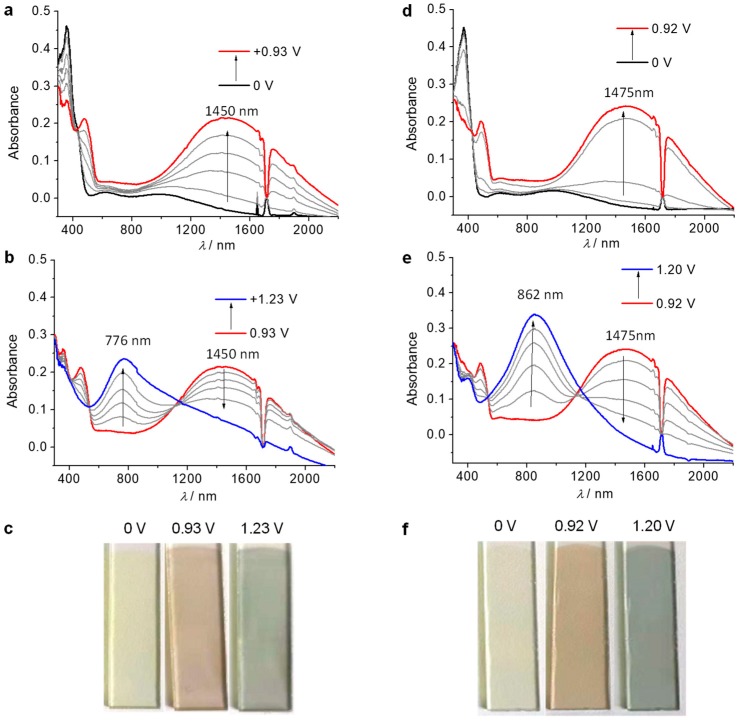
(**a**,**b**,**d**,**e**) Absorption spectral changes and (**c**,**f**) pictures at different applied potentials of (**a**–**c**) **P1**/ITO and (**d**–**f**) **P2**/ITO films recorded during spectroelectrochemical measurements. The applied potentials are referenced versus Ag/AgCl.

Spectroelectrochemistry of **P1** and **P2** films on the ITO glass electrodes were tested in dichloroethane. Figure 4 shows the changes in absorption spectra and color of **P1** and **P2** in response to different applied potentials. Two-step changes are clearly observed from these experiments. Taking **P1** as an example (Figure 4a–c), when the potential was increased from 0 V to +0.93 V, the absorption peak at 360 nm decreased continuously. During this process, two new absorption peaks appeared at 480 and 1450 nm. They are ascribed to the tetraphenylbenzidine radical cation-localized absorptions and the IVCT band, respectively. The color change of the film from pale yellow to brown was observed in this step. When the potential was further increased to +1.23 V, the IVCT absorption band at 1450 nm decreased significantly, and a new absorption peak at 776 nm was observed. The latter peak is considered to be originated from the formation of tetraphenylbenzidine dicationic species. The color of the film turned blue in the double oxidation step. Thin film **P2** also showed a similar two-step spectral and color changes (Figure 4d–f). One difference is that the new absorption peak observed during the double oxidation step located at 862 nm, which is slightly red-shifted with respect to that of **P1**.

### 3.4. Electrochromic Switching

**Figure 5:**
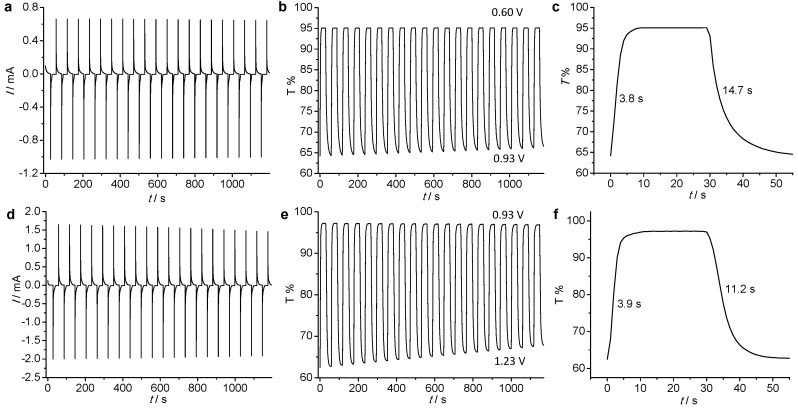
Electrochromic switching of **P1** between (**a**–**c**) +0.60 and +0.93 V vs. Ag/AgCl and (**d**–**f**) between +0.93 and +1.23 V in 0.1 M *^n^*Bu_4_NClO_4_/ClCH_2_CH_2_Cl. (**a**,**d**) Current assumption. (**b**,**c**) Transmittance changes monitored at 1450 nm. (**e**,**f**) Transmittance changes monitored at 776 nm.

**Figure 6:**
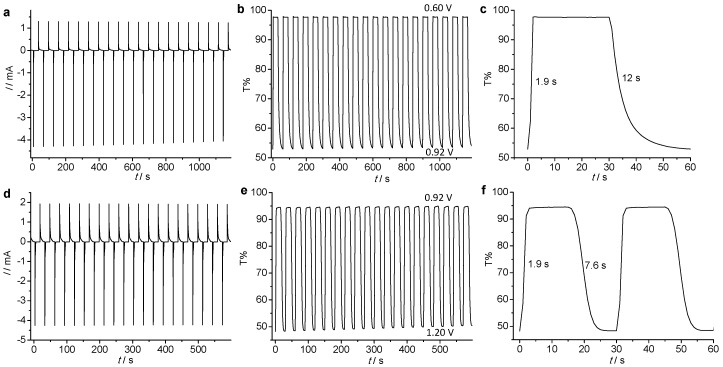
Electrochromic switching of **P2** between (**a**–**c**) +0.60 and +0.92 V vs. Ag/AgCl and (**d**–**f**) between +0.92 and +1.20 V in 0.1 M *^n^*Bu_4_NClO_4_/ClCH_2_CH_2_Cl. (**a**,**d**) Current assumption. (**b**,**c**) Transmittance changes monitored at 1475 nm. (**e**,**f**) Transmittance changes monitored at 862 nm.

The electrochromic switching properties, including the contrast ratio (∆*T*%), response time and cyclic stability of **P1** and **P2** films were further examined by double-potential step chronoamperometry (Figure 5 and Figure 6). When the potential was varied, corresponding percent transmittance (*T*%) changes of the film at a specific wavelength were monitored. When the potential was switched between +0.6 V to +0.93 V vs. Ag/AgCl (the first oxidation step), the contrast ratio of **P1** is 31% at 1450 nm and the response time was estimated to be 3.8 and 14.7 s for the reduction (bleaching, *t*_b_) and oxidation (coloring, *t*_c_) process, respectively. The response time was estimated by the duration to reach 90% of the maximum contrast ratio. The coloration efficiency (CE) for this process was calculated to be 390 cm^2^/C according to CE(λ) = ΔOD/*Q*_d_ = log[*T*_b_/*T*_c_]/*Q*_d_ (*Q*_d_ is the charge density in C/cm^2^; *T*_b_ and *T*_c_ are the transmittance values in the bleached and colored states, respectively). As the applied potential was switched between +0.93 to +1.23 V (the second oxidation step), the transmittance of **P1** was monitored at 776 nm. In this process, we achieved a contrast ratio of 35% with a CE value of 210 cm^2^/C at 776 nm. The response time is 3.9 and 11.2 s for the bleaching and coloring step, respectively (Figure 5).

The **P2** film showed slightly better electrochromic properties than **P1**. During the first oxidation step, a contrast ratio of 45% at 1475 nm was achieved with *t*_b_ = 1.9 s, *t*_c_ = 12 s, and CE = 440 cm^2^/C. During the second oxidation step, it exhibited a ∆*T*% of 46% at 862 nm with *t*_b_ = 1.9 s, *t*_c_ = 7.6 s, and CE = 300 cm^2^/C (Figure 6). In addition, **P2** shows a better cyclic stability than **P1**. After 50 cycles potential switching in the first oxidation step, the contrast ratio of **P1** at 1450 nm dropped from 31% to 25% (around 20% drop of the original performance). In contrast, the contrast ratio of **P2** at 1475 nm only dropped 4% of its original value (from 45% to 43%) after the similar 50 cycles of switching (Figure S3).

## 4. Discussion

Polymers **P1** and **P2** show similar electrochemistry and absorption spectral changes upon stepwise oxidations. In contrast, P**2** exhibits slightly better electrochromic properties than **P1**, including higher contrast ratio, shorter response time, and better cyclic stability. Both polymers are believed to have a polymeric network connected by the redox-active tetraphenylbenzidine bridge. Compound **2** has a longer side arm than **1**. As a result, **P2** should have a more porous structure than **P1**, which will facilitate the counter ion diffusion during the heteogenerous oxidation and reduction processes. This may be responsible for the observed faster response of **P2** versus **P1**. It is unknown at this stage whether the pyrene framework is involved in the electron transfer processes. In addition, the polymerization of compound **2** with a longer sidearm may lead to some entangled or interdigitated structure, which could enhance the cyclic stability of P**2**. The electrochromic contrast ratio of **P1** and **P2** is in the range of 30% to 50%, which is comparable to known NIR electrochromic materials at a similar wavelength. For instance, the electrochromic ruthenium materials reported by Wang and coworkers displayed a contrast ratio of around 40% at 1550 nm [15]. In addition, Xu and coworkers reported electrochromism of isoindigo polymers with a contrast ratio range of 59% at 1500 nm [19].

In the first step electrochromism, both films display electrochromism at the very similar wavelength around 1500 nm, which is the typical wavelength of the IVCT of tetraphenylbenzidine [30,35]. This suggests the presence of the pyrene does not distinctly affect the NIR absorption. The role of pyrene is simply to act as a multi-branched unit to form a polymeric network. In the second step, the electrochromic wavelength of **P2** is about 90 nm red-shifted with respect to that of **P1**. The red-shift is possibly caused by the increased π-conjugation of the sidearm group of **P2** with respect to that of **P1**.

## 5. Conclusions

In conclusion, thin films of two pyrene-cored multi-triphenylamine derivatives have been prepared by oxidative electropolymeriztion. These films show two consecutive redox waves and two-step electrochromism in the NIR region with moderate contrast ratio. In contrast, the polymeric film prepared from the monomer with longer side-arms exhibits better electrochromic properties, including higher contrast ratio, shorter response time, and better cyclic stability, making it potentially useful in optic telecommunication. This information is also important for the molecular design of electrochromic materials.

## Supplementary Materials

The following are available online at http://www.mdpi.com/2073-4360/11/1/73/s1, Figure S1. CVs recorded during the oxidative electropolymerization of **1** and **2** at different potential regions. Figure S2. SEM images of **P1**/ITO glass film. Figure S3. Transmittance changes of **P1** and **P2** after 50 cycles of double-potential switching. Figure S4. ^1^H NMR of compound **1**; Figure S5. ^1^H NMR of compound **2**; Figure S6. HRMS data of compound **1**; Figure S7. HRMS data of compound **2**.
