# Supplementary material for: Electrochromism in Electropolymerized Films of Pyrene-Triphenylamine Derivatives
Source: Polymers (Basel). 2019 Jan 5;11(1):73. doi: 10.3390/polym11010073 (PMC6402011; doi:10.3390/polym11010073)
Supplement: Supplementary file 1 [file polymers-11-00073-s001.pdf]

Supplementary Materials

Electrochromism in electropolymerized films of pyrene-triphenylamine derivatives

Tian-Ge Sun [1,2], Zhi-Juan Li [1,2], Jiang-Yang Shao [1], and Yu-Wu Zhong [1,2,*]

[1] Key Laboratory of Photochemistry, Beijing National Laboratory for Molecular Sciences, CAS Research/Education Center for Excellence in Molecular Sciences, Institute of Chemistry, Chinese Academy of Sciences, Beijing 100190, China

[2] School of Chemical Sciences, University of Chinese Academy of Sciences, Beijing 100049, China

* Correspondence: zhongyuwu@iccas.ac.cn

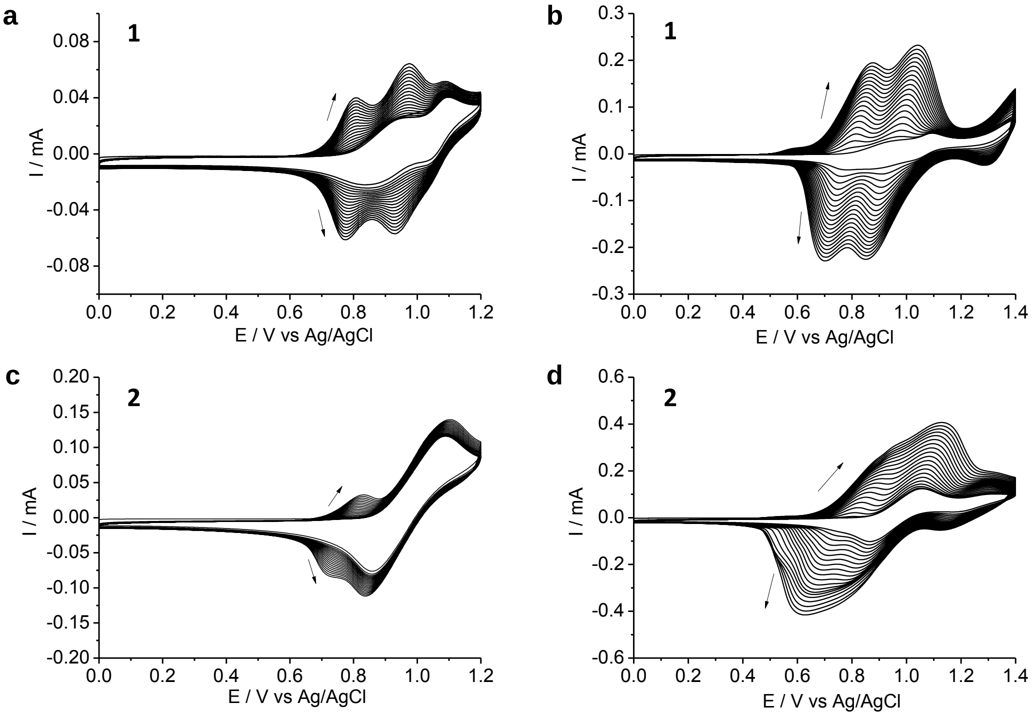

Figure S1. CVs recorded during the oxidative electropolymerization of (a,b) **1** and (c,d) **2** on an ITO glass electrodes by 20 repeated potential scan cycles between (a,c) 0 and +1.2 V or (b,d) 0 and +1.4 V at 100 mV/s.

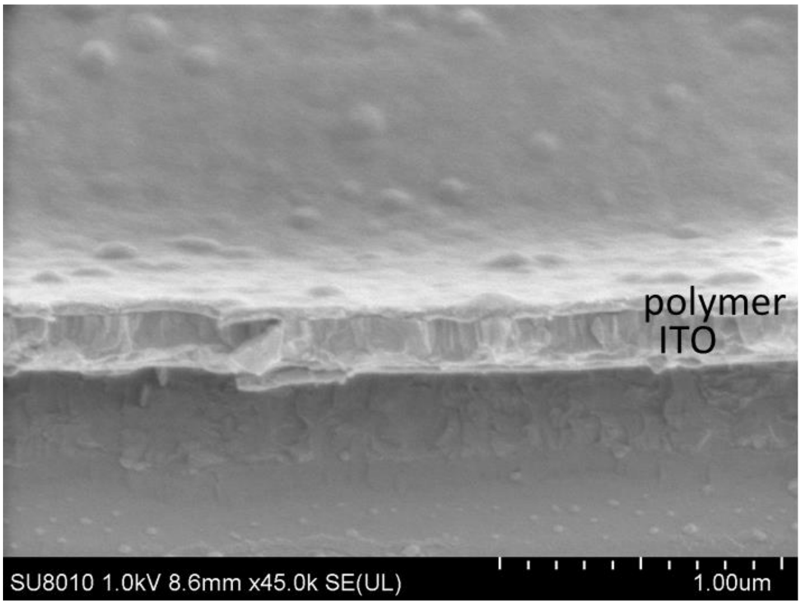

Figure S2. SEM images of **P1**/ITO glass film. Images were obtained using a field-emission microscope (JEOL S-4800) operated at an acceleration voltage of 1 kV. Prior to measurement, an ultrathin conductive Au coating was deposited on the top of the polymeric films on ITO glass electrodes by low vacuum sputter coating of the sample.

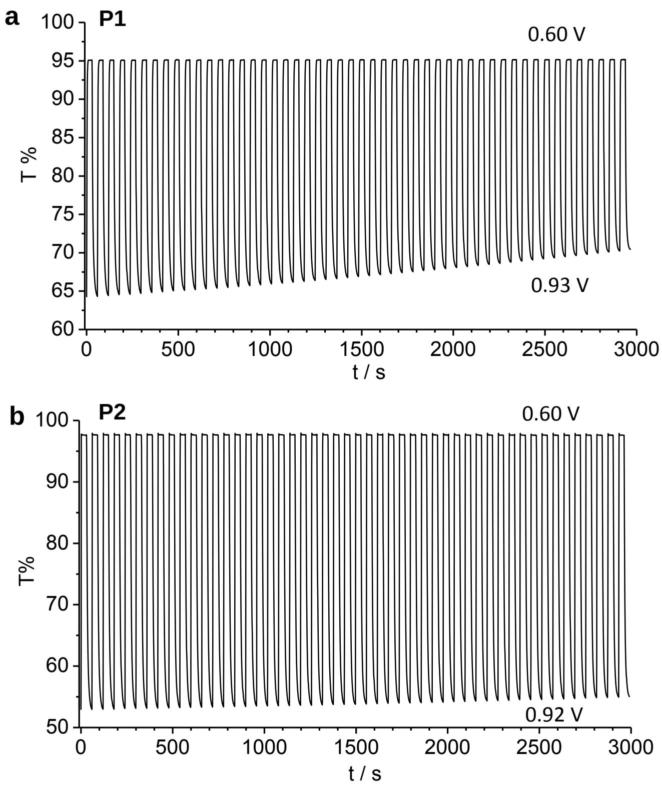

Figure S3. Transmittance changes of (a) **P1** at 1450 nm switched between +0.60 and +0.93 V and of (b) **P2** at 1475 nm between +0.60 and +0.92 V in 0.1 M $^{n}Bu_4NClO_4/ClCH_2CH_2Cl$.

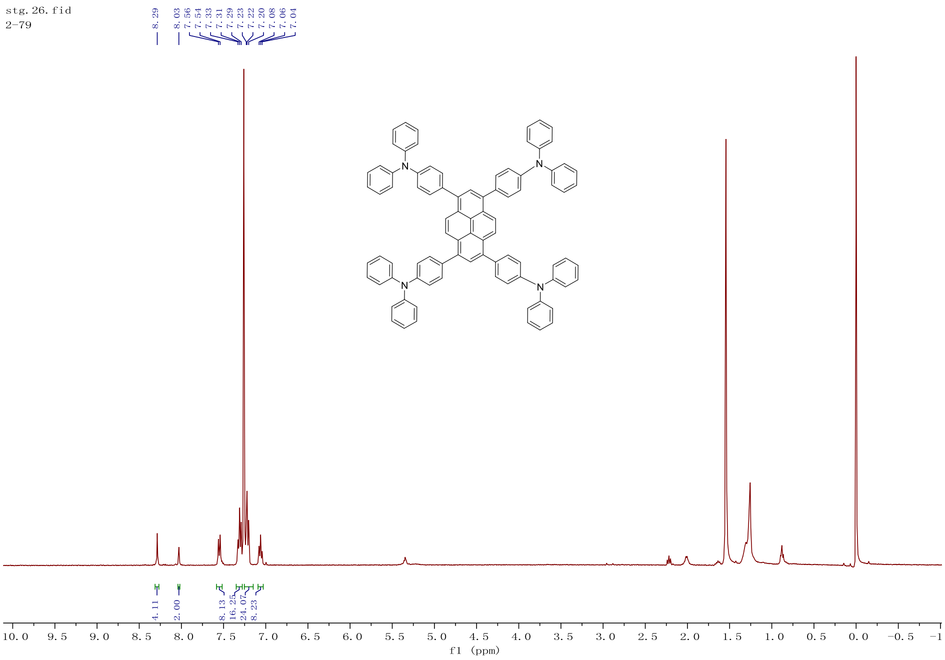

Figure S4. ^{1}H NMR spectrum of compound **1** (400 MHz, $CDCl_3$, 298 K).

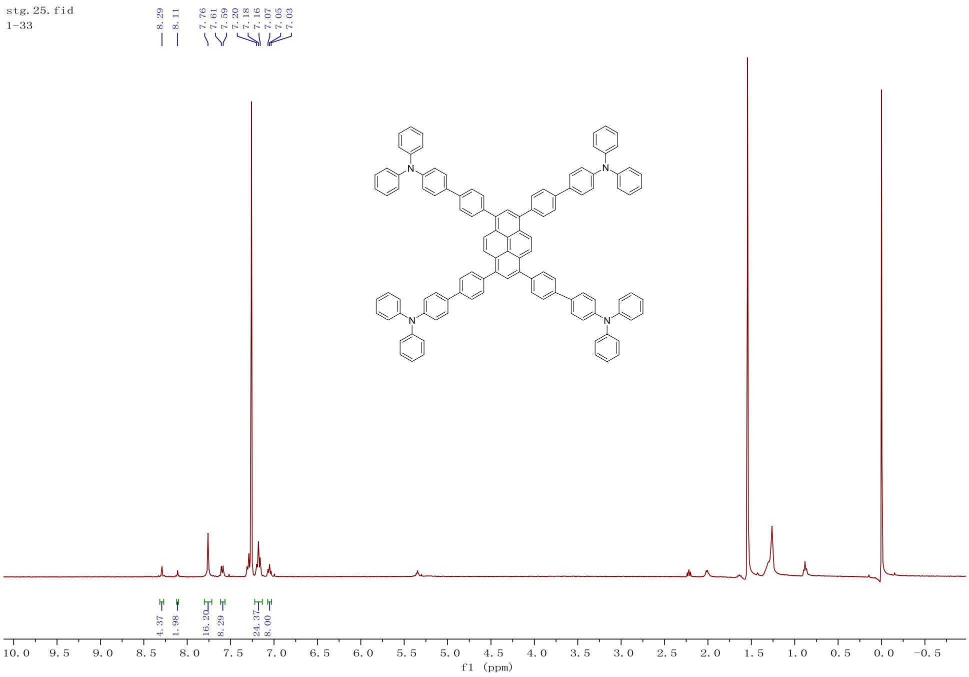

Figure S5. ^{1}H NMR spectrum of compound **2** (400 MHz, $CDCl_3$, 298 K).

MALDI,2-79,20181023

Analysis Info

Analysis Name D:\Data\MALDI\2018\1023\2-79_0_M16_000001.d
Method MALDI_P_100-3000
Sample Name MURU-N-ESI
Comment

Acquisition Date 10/23/2018 6:05:22 PM
Operator
Instrument solariX

Acquisition Parameter

Acquisition Mode	Single MS	Acquired Scans	4	Calibration Date	Mon Oct 22 05:40:57
Polarity	Positive	No. of Cell Fills	1	Data Acquisition Size	2098152
Broadband Low Mass	101.1 m/z	No. of Laser Shots	10	Data Processing Size	4194304
Broadband High Mass	2800.0 m/z	Laser Power	28.0 lp	Apodization	Sine-Bell Multiplication
Source Accumulation	0.001 sec	Laser Shot Frequency	0.020 sec		
Ion Accumulation Time	0.300 sec				

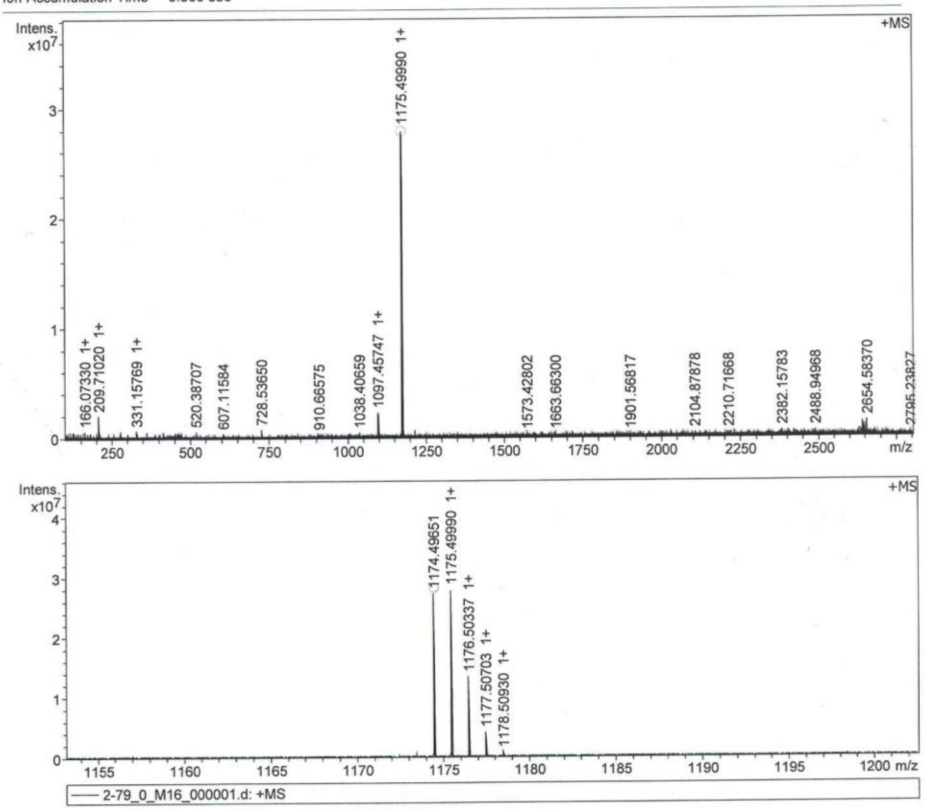

Meas. m/z	#	Ion Formula	Score	m/z	err [ppm]	Mean err [ppm]	mSigma	rdb	e⁻ Conf	N-Rule
1174.496510	1	C88H62N4	100.00	1174.496899	0.3	0.2	15.4	60.0	odd	ok

Figure S6. HRMS data of compound **1**.

MALDI,1-33,20181023

Analysis Info

Analysis Name	D:\Data\MALDI\2018\1023\1-33_0_M15_000002.d	Acquisition Date	10/23/2018 6:04:22 PM
Method	MALDI_P_100-3000	Operator	
Sample Name	MURU-N-ESI	Instrument	solariX
Comment			

Acquisition Parameter

Acquisition Mode	Single MS	Acquired Scans	4	Calibration Date	Mon Oct 22 05:40:57
Polarity	Positive	No. of Cell Fills	1	Data Acquisition Size	2098152
Broadband Low Mass	101.1 m/z	No. of Laser Shots	10	Data Processing Size	4194304
Broadband High Mass	2800.0 m/z	Laser Power	28.0 lp	Apodization	Sine-Bell Multiplication
Source Accumulation	0.001 sec	Laser Shot Frequency	0.020 sec		
Ion Accumulation Time	0.300 sec				

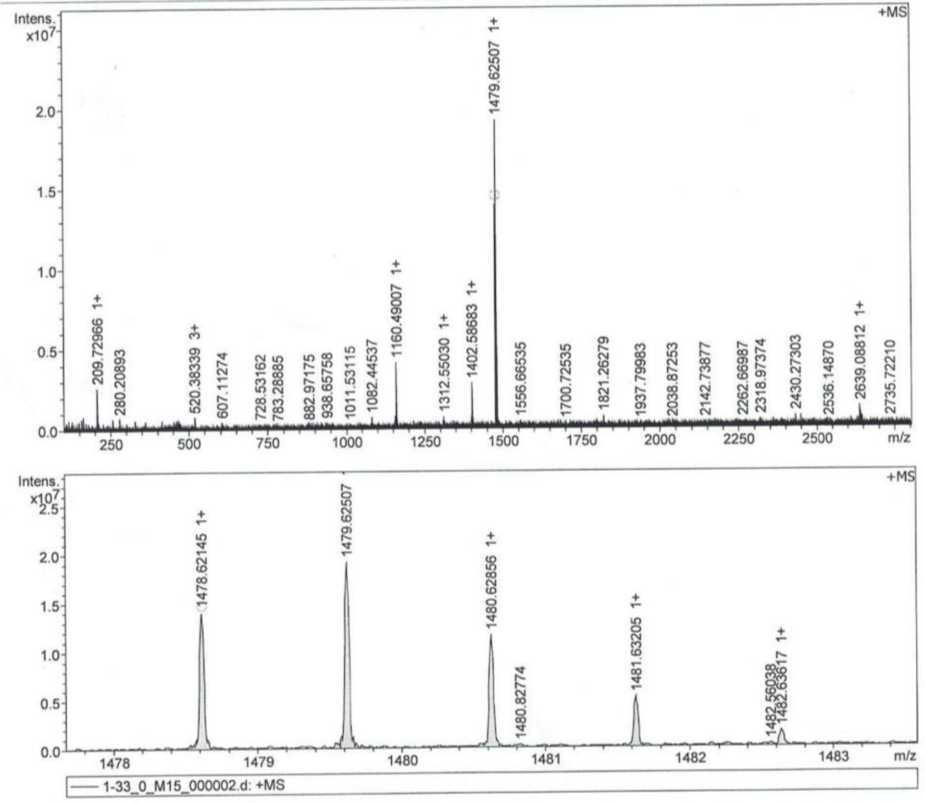

Meas. m/z	#	Ion Formula	Score	m/z	err [ppm]	Mean err [ppm]	mSigma	rdb	e⁻ Conf	N-Rule
1478.621450	1	C112H78N4	100.00	1478.622100	0.4	0.2	31.5	76.0	odd	ok

Figure S7. HRMS data of compound **2**.
